# Academic pharmacist competencies in ordinary and emergency situations: content validation and pilot description in Lebanese academia

**DOI:** 10.1186/s12909-023-04712-4

**Published:** 2023-10-06

**Authors:** Jihan Safwan, Marwan Akel, Hala Sacre, Chadia Haddad, Fouad Sakr, Aline Hajj, Rony M. Zeenny, Katia Iskandar, Pascale Salameh

**Affiliations:** 1 School of Pharmacy, Lebanese International University, Beirut, Lebanon; 2 INSPECT-LB (Institut National de Santé Publique, d’Épidémiologie Clinique Et de Toxicologie-Liban), Beirut, Lebanon; 3 School of Medicine, Lebanese American University, Byblos, Lebanon; 4 School of Health Sciences, Modern University of Business and Science, Beirut, Lebanon; 5 Research Department, Psychiatric Hospital of the Cross, Jal El Dib, Lebanon; 6 Faculté de Pharmacie, Université Laval, Québec, Canada; 7 Oncology Division, CHU de Québec Université Laval Research Center, Québec, Canada; 8 Department of Pharmacy, American University of Beirut Medical Center, Beirut, Lebanon; 9 Epidemiology and Biostatistics Department, Faculty of Public Health II, Lebanese University, Beirut, Lebanon; 10 Faculty of Pharmacy, Lebanese University, Hadat, Lebanon; 11 University of Nicosia Medical School, Nicosia, Cyprus

**Keywords:** Academic pharmacists, Academic competencies, Competency framework, Developing country, Lebanon, Specialized competencies

## Abstract

**Background:**

In the absence of a similar study in the Lebanese context, this study aimed to validate the content of the specialized competencies frameworks of academic pharmacists (educators, researchers, and clinical preceptors) and pilot their use for practice assessment in the context of multiple severe crises.

**Methods:**

A web-based cross-sectional study was conducted between March and September 2022 among academic pharmacists enrolled by snowball sampling using a questionnaire created on Google Forms.

**Results:**

The suggested frameworks had appropriate content to assess the competencies of academic pharmacists. Educators and clinical preceptors were confident in all their competencies except for emergency preparedness. Researchers had varying levels of confidence, ranging from moderate to high confidence for many competencies, but gaps were reported in fundamental research, conducting clinical trials, and pharmacy practice research (mean < 80). Educators and researchers relied primarily on experience and postgraduate studies, while clinical preceptors emphasized undergraduate studies to acquire their respective competencies. Continuing education sessions/programs were the least cited as a competency-acquiring venue across all roles.

**Conclusion:**

This study could develop and validate the content of frameworks for specialized competencies of academic pharmacists, including educators, researchers, and clinical preceptors, in a challenging setting. The frameworks were also piloted for practice assessment, which could contribute to supporting effective performance and sustained development of practitioners and help link the skills and competencies pharmacists learn during their studies with those required for a career in academia.

**Supplementary Information:**

The online version contains supplementary material available at 10.1186/s12909-023-04712-4.

## Introduction

Academic pharmacists play a crucial role in maintaining the quality of pharmacy education. Competent educators are essential to improving education quality, outcomes, and impact [1], and a lack of competence among educators can adversely affect education quality. Therefore, it is essential to define the competencies necessary to improve pharmacy education quality so that academic staff has and continually improves these competencies through continuing professional development [2]. Understanding these competencies allows for an efficient evaluation of academic staff members while helping them self-reflect, assess their professional needs, and set their goals. Proper handling of these competencies leads to well-rounded educators, just as addressing all aspects of student competencies produces well-rounded learners [3]. Similar reasoning would also apply to pharmaceutical researchers.

Pharmacists in academia face challenges when performing their duties as educators, researchers, and clinical preceptors. Furthermore, the mismatch described in the literature between their formal education and the demands of their multidimensional leadership roles might become more pronounced as they take on increasing administrative responsibilities throughout their careers [4–6]. While pharmacy schools develop standard academic programs to educate students in clinical knowledge, competencies, and other skills needed for patient care [7], the success of educational healthcare institutions relies heavily on defined career plans for the advancement of faculty members [8, 9]. The academic administration is responsible for helping their faculty members develop the necessary competencies to carry out these plans and achieve the institutional strategic goals [10].

Competency-based developmental frameworks are widely used to standardize the necessary competencies for a given role and determine the requirements for education, training, and career advancement. They are becoming more prevalent in health professions in high-income countries [11] and include a set of behavioral competencies that helps practitioner development and facilitates effective and long-lasting performance. An example is the evidence-based Global Competency Framework for Educators and Trainers in Pharmacy (FIP-GCFE) created by the International Pharmacy Federation (FIP) as a new instrument to enhance the professional development of pharmacists working in any area of pharmaceutical education [12]. This framework, which comprises several competency clusters (i.e., education, training, and development; research, evaluation, and scholarship; expert professional practice; working with others; management, strategy, and planning; and leadership), is an invaluable resource that helps pharmacy educators and trainers at the individual, corporate, national, and international levels maintain and improve their practice continuously. Such a framework could be adapted in the form of a questionnaire to assist in identifying the relevance and applicability of the framework in a specific national and local setting [2, 12].

Moreover, pharmacists have a long history of involvement in public health emergency preparedness and response, including prevention through periodic immunizations and guaranteeing access to medications [13, 14]. Recently, pharmacy professionals (including academic pharmacists) responded quickly to the COVID-19 pandemic by collaborating with other healthcare professionals, official authorities and departments, professional associations, boards of pharmacy, non-profit organizations, and the private sector to provide necessary services [15]. The abovementioned deeds demonstrate the responsibility of pharmacists in all public health areas, including emergencies such as COVID-19, as outlined in policy statements and guidelines released by American Public Health Association (APHA) and FIP [16–19]. Of note, this aspect was not included in the recently released Global Competency Framework for Pharmacy Educators [2, 12].

In Lebanon, a developing country affected in the three previous years by a severe sanitary and socioeconomic crisis, 8855 pharmacists work in community pharmacies (63.4%), hospital settings (5%), academia (3.8%), industry (24%), and other areas of practice (3.7%), as per the figures from 2018 [20]. The number of pharmacy researchers graduating each year in Lebanon is limited because the authorities do not recognize research degrees as a specialty [21], and universities prioritize teaching and service-related activities over research for hiring, promoting, and remunerating [22]. Also, a relatively small number of pharmacists hold master's or doctoral degrees, further explaining the decline in confidence in the research skills acquired through graduate studies [23]. Hence, although pharmacists in academia account for a small percentage of the workforce, they play a crucial role in educating and preparing pharmacy students to become competent healthcare professionals [24] and advancing pharmaceutical knowledge through research projects. Therefore, an adequate framework is necessary for academics to assess their learning needs and provide a ground for personal advancement, continuing education, and professional development objectives [2], particularly in the challenging Lebanese context. A robust education and training program must also be available to ensure that academic pharmacy professionals have the tools to produce knowledgeable pharmacists who are competent in delivering pharmaceutical healthcare [25, 26].

Thus, this study aimed to validate the content of the specialized competencies of academic pharmacists and pilot three frameworks (educators, researchers, and clinical preceptors) for practice assessment in the context of multiple severe crises.

## Methods

### Description of the process

In January 2021, the Order of Pharmacists of Lebanon (OPL, the official pharmacists’ association in Lebanon), in collaboration with the National Institute of Public Health, Clinical Epidemiology, and Toxicology-Lebanon (INSPECT-LB), endeavored to define the competencies of academic pharmacists and formulate a competency model that encompasses all the roles of academic pharmacists to provide structural support for the provision of education and continuous professional development in the pharmacy career. This model would allow pharmacists in academia to evaluate and improve their capabilities, thereby supporting academics at all levels in acquiring and honing the necessary competencies for a skilled and professional workforce.

The literature was thoroughly sought to identify existing structures, but no validated framework related to academic pharmacists was found at this date. Hence, a competency framework for academic pharmacists was to be developed, drawing inspiration from several studies; it covered three roles, i.e., teaching, research, and clinical preceptorship. Educator competencies included designing and planning learning programs, teaching and facilitating learning, assessing student learning, creating effective and innovative learning environments, providing student guidance, and engaging in professional development [5]. As for researchers, their competencies should encompass soft skills related to critical thinking/scientific inquisitiveness/ethical thinking, research conduct/strategic thinking, oral and written communication/grantsmanship, interprofessional collaboration and teamwork/leadership, project management (including planning and time management), and information technology [22]. Lastly, clinical preceptors should focus on ethical and professional skills, supervising and mentoring students, facilitating learning and coaching, time management, interpersonal communication, cultural competency, leadership skills, motivation skills, knowledge, and research management [27].

Whatever the role of academic pharmacists, they are all required to have competencies related to emergency preparedness [16–19]. Since the current assessment was carried out in the context of multiple crises (socioeconomic and sanitary) in Lebanon, competencies relevant to this aspect have been added, including responses to predicaments, operation management during emergencies, patient care and population health interventions and evaluation, research, and dissemination of results for impact and outcomes. By critically reflecting on these competencies, pharmacy educators, researchers, and clinical preceptors could better equip themselves to handle the ongoing COVID-19 pandemic and upcoming public health emergencies, in addition to the multifaceted crises the country is facing. These competencies and their associated behaviors could also serve as a tool for self-assessment, retention strategies, career advancement, and performance evaluation in practice settings.

### Source of the three competency frameworks

The three frameworks were based on the OPL previously suggested frameworks for academic pharmacists: one for educators (classroom teaching), one for researchers, and a third one for clinical preceptors [22, 27, 28]. The section related to emergency preparedness was added to the frameworks previously suggested by the OPL and adapted to the role played by the academic pharmacist [15, 17–19].

### Content validation of the three frameworks

Six academic pharmacy experts, all of whom are researchers, with five being educators and four being clinical preceptors, assessed the content of the three frameworks. A Delphi technique was applied to agree by more than 90% on the suggested items until reaching a consensus [29, 30]. The final frameworks were finalized and shared on the official social media pages (Facebook, Instagram) of the OPL and specific WhatsApp groups of registered pharmacists. The OPL frequently uses those social media platforms to disseminate messages.

### Piloting the three competency frameworks

The three frameworks were distributed to all full-time academic pharmacists in the five universities teaching pharmacy in Lebanon (overall estimated *n* = 60). Pharmacists completed the questionnaire according to their role in academia (educator, researcher, and/or clinical preceptor). Before filling out the questionnaire, participants were briefed about the topic and the different elements of the survey, including the voluntary aspect, and were assured of the anonymity of the data collection process. Each framework required 30 min to be completed.

### Pilot study design

A web-based cross-sectional study was conducted between March and September 2022 among academic pharmacists enrolled by snowball sampling using a questionnaire created on Google Forms. To ensure that the sample is representative of the demographic characteristics of all pharmacy academicians in Lebanon, data were collected from all the schools of pharmacy in the country.

### Ethical aspect

The Lebanese International University Research and Ethics Committee approved the project (Approval number: 2020RC-063-LIUSOP). Written informed consent was obtained from each participant at the beginning of the survey.

### Sample size calculation

The minimum sample size was calculated using the Centers for Disease Control (CDC) Epi-info software. The expected frequency was set at 90% since the specialized competencies and domains were expected to be demonstrated by pharmacists working in academia. Accordingly, based on a total of 60 full-time academic pharmacists, a minimum sample of 37 participants was required, considering an acceptable error of 6% and a 95% confidence interval, with a 5% alpha error and a power of 80%. Data collection was stopped when 38 academic pharmacists filled out the frameworks.

### Description of the questionnaires

The questionnaires were developed in English since this language is commonly spoken in Lebanon by healthcare professionals, including pharmacists in academia, and comprised four sections (Supplementary Files 1, 2, and 3).

- Section 1 collected the sociodemographic, educational, and professional characteristics. In this part of the questionnaire, participants were asked about their general sociodemographic data, including their age, gender, nationality, area of work, university of graduation, highest educational level, years of experience, and the number of days of work per week.
- Section 2 consisted of domains, including competencies and behaviors (1 question per item), related to academic pharmacists (educators, researchers, and clinical preceptors). Behaviors were assessed on a 5-point Likert scale, ranging from highly confident (5 points) to fairly confident (4 points), not sure/I do not know (3 points), slightly confident (2 points), and not confident at all (1 point). Competency scores were calculated by summing the answers to items (behaviors); responses were standardized over one hundred for ease of comparison. This section varied according to the role played by the academic pharmacist.
- Section 3 included questions related to preparedness and response to emergencies (i.e., emergency preparedness; operation management during emergencies; patient care and population health interventions and evaluation; research; and dissemination for impact and outcomes). This section was tailored to fit each role.
- Section 4 inquired about the context in which the competencies were acquired, including undergraduate studies, postgraduate degrees, continuing education sessions/programs, and professional experience.

### Statistical analysis

The data were analyzed using SPSS software version 25. A descriptive analysis was done using counts and percentages for categorical variables, while means (M) and standard deviations (SD) and medians and interquartile ranges (IQR) were presented for continuous measures. This procedure was performed for all academic pharmacists and the strata of the sample (educators, researchers, and clinical preceptors).

Bivariate analysis was conducted using nonparametric tests since the normality of continuous variables was not ensured: The Mann–Whitney test was used for comparing two groups, and ANOVA was used to compare three groups or more. A p-value of less than 0.05 was considered significant.

## Results

### Piloting outcome

A total of 38 academic pharmacists responded to the survey, distributed as follows: 19 educator pharmacists (classroom teachers), 9 research pharmacists, and 10 clinical preceptors. The piloting of the developed frameworks yielded satisfactory results among these pharmacists. Questions were answered by all participants, and no answers were missing. Thus, no items were removed from the list of behaviors.

Educator competencies were six and comprised: designing and planning learning programs (5Q), teaching and supporting learning (4Q), assessing student learning (4Q), creating effective/innovative learning environments (7Q), providing student guidance (5Q), and professional development (7Q).

Researcher competencies were thirteen: soft skills (11Q), communication (4Q), interprofessional collaboration (4Q), project management (3Q), fundamental sciences (3Q), information technology skills (1Q), conducting fundamental research (5Q), translational clinical research (3Q), trial analysis skills (4Q), conducting clinical trials (9Q), evaluating clinical research (7Q), pharmacy practice research (9Q), and professional development (2Q).

Regarding clinical preceptors, the competencies assessed were ten: ethical and professional skills (9Q), supervising and teaching students (8Q), facilitating learning and coaching (8Q), time management (15Q), interpersonal communication (8Q), cultural competency (8Q), leadership skills (9Q), motivation skills (4Q), knowledge (3Q), and research management (2Q).

As for the emergency preparedness section, the number of questions varied per role, including 13Q for educators, 20Q for researchers, and 17Q for clinical preceptors.

### Characteristics of the pilot sample

The sociodemographic, educational, and professional characteristics of academic pharmacists are presented in Table 1. The mean age of our population was 36.79 ± 8.53 years, with slightly more aged participants among researcher pharmacists. Most participants were females (74%), with very few males among researchers (11.1%). The vast majority had a Doctor of Pharmacy (PharmD) (79%), while master’s degrees (89%) and Doctor of Philosophy (PhD) (56%) were more common among researchers. PharmD was the highest level of education reported by half of the sample. The mean experience duration was 7.29 ± 7.54 years, slightly more among research pharmacists (9.33 ± 9.89 years). Since all participants were full-timers, the vast majority worked 4 to 5 days per week. All the universities teaching pharmacy were represented in the sample.

**Table 1:** Characteristics of academic pharmacists

Characteristic	Total Academic Pharmacists *N* = 38	Sample 1: Educator pharmacists *N* = 19	Sample 2: Research pharmacists *N* = 9	Sample 3: Clinical preceptors *N* = 10
**Age M(SD)**	36.79 (8.53)	36.63 (7.60)	40.33 (10.84)	33.90 (7.56)
**Median (IQR)**	38.50 [28.75–41]	38 [30–41]	41.00 [31.50–50.0]	31.50 (27.75–39.50)
**Gender**				
Female	28 (73.7%)	14 (73.7%)	8 (99.9%)	6 (60%)
Male	10 (26.3%)	5 (26.3%)	1 (11.1%)	4 (40%)
**Degrees earned**				
BS Pharm	35 (92.1%)	17 (89.5%)	8 (88.9%)	10 (100%)
PharmD/DPharm	30 (78.9%)	16 (84.2%)	4 (44.4%)	10 (100%)
Master’s	20 (52.6%)	7 (36.8%)	8 (88.9%)	5 (50%)
PhD	13 (34.2%)	6 (31.6%)	5 (55.6%)	2 (20%)
**Highest degree**				
PhD	12 (31.6%)	5 (26.3%)	5 (55.6%)	2 (20%)
PharmD/DPharm	19 (50.0%)	10 (52.6%)	2 (22.2%)	7 (70%)
Master’s or BS Pharm	7 (18.4%)	4 (21.1%)	2 (22.2%)	1 (10%)
**University of pharmacy graduation**				
BAU	4 (10.5%)	2 (10.5%)	2 (22.2%)	0
LAU	10 (26.3%)	6 (31.6%)	3 (33.3%)	1 (10%)
LIU	14 (36.8%)	7 (36.8%)	0	7 (70%)
LU	5 (13.2%)	1 (5.3%)	3 (33.3%)	1 (10%)
USJ	2 (5.3%)	1 (5.3%)	1 (11.1%)	0
Other	3 (7.9%)	2 (10.5%)	0	1 (10%)
**Mean experience M(SD)**	7.29 (7.54)	7.58 [7.22]	9.33 (9.89)	4.90 (5.51)
**Median [IQR]**	4 [2–18]	6 [1–14]	5 [1.5–20]	1 [1–9.25]
**Working days/week**				
** M(SD)**	4.45 (1.41)	4.37 (1.42)	4.44 (1.74)	4.60 (1.17)
** Median ([IQR]**	5 [4, 5]	5 [4, 5]	5.00 [4.50–5.00]	5 [4.5–5]

PhD (Doctor of Philosophy); PharmD/DPharm (Doctor of Pharmacy); BS Pharm (Bachelor of Science in Pharmacy); BAU (Beirut Arab University); LAU (Lebanese American University); LIU (Lebanese International University); LU (Lebanese University); USJ (Saint Joseph University of Beirut)

Table 2 shows the results of the competency assessment. Educator pharmacists felt confident in fulfilling all competencies (mean grade more than 90), except for emergency preparedness, where they felt less confident (mean grade of 82 out of 100). Researchers had high confidence in competencies such as communication, interprofessional collaboration, and project management (mean > 90) and moderate confidence in others (emergency preparedness, professional development, soft and information technology skills, clinical research, translational research, and trial analysis skills); they were the least confident in fundamental research, conducting clinical trials, and pharmacy practice research (mean < 80). As for clinical preceptors, they felt highly confident in all the competencies, with a mean of more than 90 for all.

**Table 2:** Self-declared competencies by academic role

**Competencies of teaching pharmacists (/total points)**	**M(SD)**	**Standardized mean (100)**	**Median [IQR]**
1 Design and plan learning (/25)	23.05 (2.63)	92.20	25 [21–25]
2 Teach and support learning (/20)	18.63 (2.17)	93.15	20 [17–20]
3 Assess student learning (/20)	18.63 (1.57)	93.15	19 [18–20]
4 Create effective/innovative learning environments (/35)	32.58 (2.85)	93.08	34 [30–35]
5 Provide student guidance (/25)	23.42 (2.06)	93.68	24 [22–25]
6 Professional development (/35)	32.32 (2.98)	92.34	33 [30–35]
7 Emergency preparedness (/65)	53.00 (9.60)	81.54	53 [52–60]
**Competencies of research pharmacists (/total points)**	**M(SD)**	**Standardized mean (100)**	**Median [IQR]**
1 Soft skills (/55)	47.78 (8.69)	86.87	47 [43.5–55]
2 Communication (/20)	18.11 (1.69)	90.55	18 [17–19.5]
3 Interprofessional collaboration (/20)	19.44 (1.13)	97.20	20 [19, 20]
4 Project management (/15)	13.78 (0.97)	91.87	14 [13–14.5]
5 Fundamental sciences (/15)	11.11 (2.37)	74.06	11 [9–13]
6 Information Technology skill (/5)	4.22 (0.44)	84.40	4 [4–4.5]
7 Conduct fundamental research (/25)	19.22 (3.15)	76.88	20 [16–21]
8 Translational clinical research (/15)	12.22 (2.73)	81.47	12 [11.5–14.5]
9 Trial analysis skills (/20)	17.44 (2.70)	87.20	19 [15.5–19.5]
10 Conducting clinical trials (/45)	35.66 (7.94)	79.24	37 [32.5–40.5]
11 Evaluation clinical research (/35)	29.89 (26.50)	85.40	29 [26.5–35]
12 Pharmacy practice research (/45)	34.78 (8.17)	77.29	36 [27–42]
13 Professional development (/10)	8.89 (1.76)	88.90	10 [7–10]
14 Emergency preparedness (/100)	85.67 (16.34)	85.67	92 [77–97]
**Competencies of clinical preceptors (/total points)**	**M(SD)**	**Standardized mean (100)**	**Median [IQR]**
1 Ethical and Professional Skills (/45)	42.70 (3.13)	94.89	44.5 [40–45]
2 Supervise and teach (/40)	37.90 (2.81)	94.75	39 [36.25–40]
3 Facilitate learning and coach (/40)	37.70 (3.53)	94.25	40 [33.5–40]
4 Time management (/75)	71.00 (5.70)	94.67	73.5 [66.5–75]
5 Interpersonal communication (/40)	37.8 (3.12)	94.50	39 [36.5–40]
6 Cultural competency (/40)	37.5 (3.21)	93.75	39 [35–40]
7 Leadership skills (/45)	42.2 (3.77)	93.78	44 [38.25–45]
8 Motivation skills (/20)	18.7 (1.64)	93.50	19.5 [17.5–20]
9 Knowledge (/15)	13.7 (1.16)	91.33	14 [12.75–15]
10 Research management (/10)	9.20 (0.92)	92.00	9.50 [8–10]
11 Emergency preparedness (/85)	80.70 (6.88)	94.94	81.5 [74–86.7]

Table 3 displays the context where academic pharmacists declared to have acquired their skills. Overall, the professional experience was the most reported source (52%), followed by postgraduate studies, with the highest median (50%) and mean (49%). However, these numbers differed by role in academia: educators and researchers relied primarily on experience and postgraduate studies, while clinical preceptors emphasized undergraduate studies. Continuing education sessions/programs had the lowest mean and median percentages across all roles.

**Table 3:** The context of acquisition of self-declared competencies by academic pharmacists

Context of competency acquisition (in percentage)	All Academic Pharmacists *N* = 38	Sample 1: Teaching pharmacists *N* = 19	Sample 2: Research pharmacists *N* = 9	Sample 3: Clinical preceptors *N* = 10
	**M(SD)**			
Undergraduate studies	38.42 (25.58)	42.89 (27.25)	18.33 (14.58)	48 (21.63)
Postgraduate studies	48.95 (26.99)	55.00 (26.82)	35.56 (20.22)	49.50 (30.59)
Continuing education	37.24 (32.98)	47.11 (33.05)	15.56 (18.78)	38 (35.99)
Professional experience	52.26 (30.00)	59.21 (29.36)	37.78 (30.73)	52.1 (28.73)
	**Median [IQR]**			
Undergraduate studies	30 [15–60]	40 [20–70]	10 [15–25]*	45 [30–70]
Postgraduate studies	50 [25–72.5]	50 [25–80]	35 [22.5–50]	40 [23.75–82.50]
Continuing education	27.50 [10–70]	50 [10–80]	10 [5–20]	17.50 [8.75–73.75]
Professional experience	40 [30–80]	70 [30–90]	30 [15–55]	38 [30–82.50]

*p=0.017 overall, *p*=0.022 for research pharmacists vs. clinical preceptors, and *p*=0.053 for research pharmacists vs. educators

No significant differences were detected among the three groups for the shared variables (sociodemographic, educational, and professional) and acquired competencies (emergency preparedness). However, researchers relied significantly less on undergraduate studies compared to the other groups (*p* = 0.017 overall; *p* = 0.022 vs. clinical preceptors, and *p* = 0.053 vs. educators).

## Discussion

This study pioneers the evaluation of specialized competencies among academic pharmacists, including educators, researchers, and clinical preceptors, using specific frameworks. These competencies would equip academic pharmacists with the necessary knowledge, skills, attitudes, and behaviors to produce competent graduates and a capable pharmaceutical workforce. It is also the first to include emergency preparedness among the required competencies for academic pharmacists in a developing country struggling with multiple crises. Of note, the FIP released the Global Competency Framework for Educators and Trainers in Pharmacy (FIP-GCFE) in September 2022 [2, 12], after our data collection period had ended; additionally, the FIP framework addressed academic pharmacy as a whole (with no distinction of specific roles), and did not address the emergency preparedness aspect within the same framework, although that would be of primary importance in a country like Lebanon. Consequently, the results of this study would serve to pilot the frameworks for academic practice assessment in Lebanon and contribute to supporting sustained professional development in a challenging setting.

Educator pharmacists reported being confident in all competencies relating to designing and planning learning, teaching and supporting learning, creating innovative/effective learning environments, providing student guidance, and professional development. This result was anticipated, considering the skills they acquire throughout their careers through research and personal and professional development [31]. Pharmacy education involves several types of research and clinical work, with the ultimate goal of offering high-quality undergraduate and graduate health-related education, which requires establishing a pharmacy education program, improving curriculum design, teaching, and learning, and fostering sustainable, evidence-based, student-centered learning communities within educational institutions [32]. Furthermore, skilled educators can employ different learning and teaching methods and means for assessment and feedback, supplemented by the proper use of digital technologies and mentorship and evaluation strategies [33]. They can also create a supportive learning environment that ensures educational quality and is conducive to students of all specialties if the necessary credentials, expertise, materials, and resources are available [34]. As such, they play a significant role in enhancing the competencies of current students, which will influence future practice. Hence, although assessing the direct effects of education on performance can be challenging, educators should be aware of current and future practices and offer the education and training required to meet societal needs and priorities [35]. Competencies should also focus on the regular identification of learning requirements, giving educators a framework to build upon their personal development, continuing education, and professional development plans. Thus, pharmacy educators must reflect on their approaches to support students and learners in acquiring new knowledge, skills, and behaviors [12].

Unsurprisingly, pharmacy educators and researchers were less confident about emergency preparedness, likely because disaster training, education, and simulations typically focus on first responders, public health professionals, physicians, and nurses rather than pharmacists [36]. Nevertheless, various research initiatives were carried out throughout the COVID-19 pandemic and were successful in determining the level of pharmacist preparedness and areas for improvement [37–41]. Thus, continuing education is necessary to improve the emergency preparedness of academic pharmacists in Lebanon.

Pharmacy researchers confirmed having high confidence in communication, interprofessional collaboration, and project management. Those competencies are crucial in research, where good communication is necessary to build relationships within and between professions, leading to successful interprofessional collaborations and work on projects that would ultimately enhance healthcare [42]. Interprofessional collaboration acts as a catalyst for positive advancements in research by providing access to resources and enabling more in-depth and impactful investigation [43–46]. Collaboration in research can result in economic benefits and improved clinical outcomes, leading to more efficient healthcare delivery [47]. As for project management, its related competency involves the integration of technical skills, cognitive aptitude, and interpersonal ability [48] to direct activities during projects from initiation through completion to achieve the expected outcomes [49]. Within the scope of pharmacy education, researchers should demonstrate project management skills to successfully manage an educational project at the team or organization levels [2, 50]. Regarding other competencies, such as emergency preparedness, professional development, soft and information technology, clinical research, translational research, and trial analysis, researchers reported intermediate confidence, in contrast with other competencies, emphasizing the need for improvement in these areas [51]. Soft and information technology skills are of primary importance for research pharmacists working in academia, as, in addition to performing research, they must be prepared to deliver online lectures, seminars, tutorials, practical demonstrations, and learning programs. They could also be asked to represent their institution at conferences and other events [52]. Furthermore, fundamental research, conducting clinical trials, and pharmacy practice research were declared with the lowest confidence level. In fact, the majority of studies (> 90%) in Lebanon are observational, carried out mainly by academic and hospital pharmacists, while clinical trials compared account for less than 10% [51]. This result emphasizes the need to address these competencies since the translation of innovative preclinical discoveries into substantive improvements in patient care relies on clinical pharmaceutical research [53].

Our results revealed that clinical preceptors were highly confident about all the suggested competencies. In Lebanon, preceptors must hold a PharmD degree or above and have worked as pharmacists for a sufficient period in the precepting context, such as a hospital or community pharmacy, to supervise postgraduate pharmacy trainees [27]. Furthermore, preceptorship and ongoing professional development are essential for preceptors to evolve into better educators and inspire future practitioners to enhance pharmacy education by imparting their rich experience to students [54]. Hence, their experience helps them develop the necessary competencies to become knowledgeable and experienced in their field of practice and proficient at mentoring and educating students [54, 55].

It is crucial to understand the context in which the academic pharmacists stated to have acquired their skills. While clinical preceptors highly valued undergraduate studies, educators and researchers primarily relied on professional experience and postgraduate degrees. PharmD programs have facilitated the introduction of novel types of faculty and staff positions, with a focus on teaching or practice and less responsibility for traditional research, supporting their explanations and emphasizing the significance of undergraduate studies in developing the necessary skills to become a clinical preceptor [56]. Also, obtaining advanced research-based fellowships, master's, or doctoral degrees paves the way for faculty members to conduct research in biomedical, pharmaceutical, clinical, and administrative sciences within pharmacy programs [56].

Lastly, continuing education seminars and programs were the least emphasized. Although pharmacists are eager to participate in such programs [57], the lack of a formal continuing education program for pharmacists in Lebanon may hinder the ability to promote lifelong learning. It is recommended that pharmacy authorities in Lebanon create continuing professional development programs tailored to the specific roles of academic pharmacists. It is also imperative to involve all relevant stakeholders, as their efforts and support would ensure the quality of pharmacy education and workforce, leading to improved practices and, ultimately, better patient health outcomes.

### Limitations of the study

This pilot study is the foundation for further work to fully validate and assess three specialized competency frameworks among academic pharmacists in a multiple-crisis context. The sampling method might have resulted in a selection bias; information bias related to the overestimation and overconfidence of respondents is also possible. Thus, large-scale studies are necessary to assess the construct validity and reliability of the suggested frameworks and show more precise descriptive and analytical results. Furthermore, a benchmark with the FIP Global Framework is also recommended to compare the Lebanese academic pharmacists’ self-declared competencies to those deemed essential at the global level.

### Future implications

The Lebanese frameworks for specialized competencies in academic pharmacy has considerable implications for national and global policy and practice. This pioneering effort in the field is notable, as few other countries have established such specialized competencies for pharmacy educators. These frameworks could support academic pharmacists in Lebanon in their professional advancement and competence development, regardless of their area of expertise within pharmaceutical education. Similar to other specialized frameworks developed in Lebanon [58–60], it is also expected to provide a vital structure and guidance to foster and facilitate educators' contributions to high-quality education and training and become an essential resource for various stakeholders, including individual instructors, pharmacy schools, and accreditation bodies.

## Conclusion

This study validated the content of three specialized competency frameworks for academic pharmacists, including educators, researchers, and clinical preceptors, in multiple crisis settings. These frameworks, addressing the three roles of academic pharmacists, were also piloted for practice assessment, which could contribute to effective performance and sustained development of practitioners. They also help link the skills and competencies pharmacists learn during their studies with those required for a career in academia.

### Supplementary Information

**Additional file 1.**

**Additional file 2.**

**Additional file 3.**

## Data Availability

The datasets used and/or analysed during the current study are available from the corresponding author on reasonable request.

## Abbreviations

APHA: American Public Health Association
CDC: Centers for Disease Control
PharmD: Doctor of Pharmacy
PhD: Doctor of Philosophy
FIP-GCFE: Global Competency Framework for Educators and Trainers in Pharmacy
FIP: International Pharmacy Federation
IQR: Interquartile ranges
M: Means
INSPECT-LB: National Institute of Public Health, Clinical Epidemiology, and Toxicology-Lebanon
OPL: Order of Pharmacists of Lebanon
SD: Standard deviations
