# Supplementary material for: Academic pharmacist competencies in ordinary and emergency situations: content validation and pilot description in Lebanese academia
Source: BMC Med Educ. 2023 Oct 6;23:737. doi: 10.1186/s12909-023-04712-4 (PMC10559412; doi:10.1186/s12909-023-04712-4)
Supplement: Supplementary file 1 — Additional file 1. [file 12909_2023_4712_MOESM1_ESM.pdf]

Advanced Competencies for Academia Pharmacists

Dear pharmacist,

You are invited to participate in a survey about advanced competencies and skills acquired upon graduation of your highest degree related to your current field of work.

This study conducted by a group of academic researchers aims to determine the domains that need strengthening for an optimal-performing public health system.

Your participation in this study is voluntary and anonymous, and the information gathered in this 20-minute questionnaire will be treated confidentially. By completing it, you are consenting to participate in this study.

We thank you in advance for your time,

The research team.

Informed consent

Please check all the boxes to proceed to the survey

☐ I have read and understood the above information

☐ I understand that my participation is voluntary

☐ I understand that my data will be kept confidential

☐ I agree to participate in this study

DEMOGRAPHICS

1. **Age:**
2. **Gender:** ☐M ☐F
3. **Level of education:**
   ☐BS Pharmacy ☐PharmD/DPharm ☐Masters ☐PhD ☐Other:
4. **Highest degree related to your main field of work:**
   ☐BS Pharmacy ☐PharmD/DPharm ☐Masters ☐PhD ☐Other:
5. **Year of graduation from school/faculty of pharmacy:**
6. **University you graduated from as a pharmacist:**
   ☐UL ☐USJ ☐BAU ☐LAU ☐LIU ☐Other, country:
7. **University you earned your highest degree from:**
   ☐UL ☐USJ ☐BAU ☐LAU ☐LIU ☐Other, country:
8. **What is your specialty?**
9. **Language of pharmacy education:**
   ☐French ☐English ☐Other:
10. **Work Location:**
    ☐Beirut ☐Mount Lebanon ☐North Lebanon ☐South Lebanon ☐Beqaa
    ☐Currently not working
11. **Number of working days per week:**
12. **Number of working hours per day:**
13. **How long (in years) have you been teaching pharmacy?**
14. **Do you have another field of work? (Please select all that apply)**
    ☐I do not have another field of work
    ☐Community pharmacy
    ☐Clinical pharmacy
    ☐Research
    ☐Other:

ACADEMIA PHARMACIST COMPETENCIES

QUESTION: How confident are you in applying the below academic teachers' competencies?	Very confident	Fairly confident	Neither/ I don't know	Slightly confident	Not confident at all
**0 Design, plan and organize teaching and learning opportunities, courses and/or programs of study.**					
**0.1** Plan and prepare new learning activities which take into account factors including study level and context.					
**0.2** Align learning activities to the course and program learning outcomes.					
**0.3** Collaborate in the design of a range of learning activities (face to face, distance or blended) to meet learning outcomes.					
**0.4** Align formative and summative assessment with teaching strategies and subject matter to measure attainment of learning outcomes.					
**0.5** Consider learning and teaching approaches which acknowledge the needs of diverse learners.					
**1 Teach and/or support learning.**					
**1.1** Foster student-centered and interactive learning and teaching approaches through a variety of activities.					
**1.2** Engage students in creative and independent learning outside formal learning environments.					
**1.3** Integrate the development of transferable and soft skills in teaching.					
**1.4** Stimulate curiosity and interest in the subject areas that encourage life-long learning.					
**1.5** Emphasize the importance of ethical behavior in all aspects of the pharmacy profession					
**2 Assess student learning and provide feedback to ensure student advancement.**					
**2.1** Use an appropriate range of formative and summative assessment techniques.					
**2.2** Align assessment strategies to learning outcomes.					
**2.3** Design clear, unambiguous assessment criteria and communicate them electively to students.					
**2.4** Provide constructive and timely feedback to students through a range of approaches.					
**2.5** Provide constructive and timely feedback to students through a range of approaches..					
**3 Create effective and innovative learning environments to optimize learning.**					
**3.1** Develop and use inclusive formal and informal learning environments appropriate to learners' needs.					

**3.2** Ensure learners are able to access and use resources / facilities effectively to guide learning.					
**3.3** Provide opportunities for students to participate in relevant extra-curricular and co-curricular activities and support for career planning.					
**3.4** Abide by relevant codes of ethics and academic integrity.					
**3.5** Integrate subject-based research in teaching and learning in a meaningful and appropriate way.					
**3.6** Effectively integrate appropriate educational technologies in the teaching and learning process.					
**3.7** Engage students in relevant aspects of professional practice.					
**4 Provide effective student support and guidance.**					
**4.1** Support learners' transitions through provision of appropriate orientation and induction activities.					
**4.2** Provide support to learners through academic advising/tutoring.					
**4.3** Access other support and professional services as necessary.					
**4.4** Refer learners to appropriate institutional and community support as necessary.					
**4.5** Respond appropriately to learners' individual needs.					
**5 Professional development.**					
**5.1** Participate in regular professional development activities.					
**5.2** Gather information on the effectiveness of own practice through actively reflecting on feedback from a range of sources, including<br>students, to inform and enhance own practice.					
**5.3** Support the professional development of colleagues.					
**5.4** Disseminate and share own and others' successful practice and initiatives.					
**5.5** Engage with interdisciplinary initiatives and/or o professional bodies and organizations.					
**5.6** Incorporate research within own professional practice.					
**5.7** Be active in professional organizations.					
**6 Pharmacist Emergency Preparedness and Response (EPR)**	**6.1 Emergency Preparedness and Response**				
**6.1.1** Check for volunteering opportunities					
**6.1.2** Check for training opportunities					
**6.1.3** Create a course on pharmacy-related emergency preparedness					
**6.1.4** Educate students about the ongoing crisis using evidence-based information and communications, including medication supply, panic buying, at risk populations, vaccines, and testing services…					
**6.1.5** Partner with local authorities for educational activities					
**6.1.6** Follow actions and recommendations of local authorities					
**6 Pharmacist Preparedness and Response in Emergency Situations**	**6.2 Operations Management**				

**6.2.1** Monitor students and workers/assistants for symptoms					
**6.3.2** Answer EPR-related calls					
**6.3.3** Participate in interdisciplinary training to EPR teams					
**6 Pharmacist Preparedness and Response in Emergency Situations**	**6.4 Evaluation, Research, and Dissemination for Impact and Outcomes**				
**6.4.1** Participate in research and studies on EPR					
**6.4.2** Publish and/or disseminate findings					
**6.4.3** Combat misinformation by disseminating evidence-based information to patients and sharing it on social media					
**6.4.4** Develop training programs to students, peers, and other staff workers					

1. **What percentage of these competencies did you acquire during your undergraduate studies?**
2. **What percentage of these competencies did you acquire during your postgraduate studies?**
3. **What percentage of these competencies did you acquire from continuing education sessions?**
4. **What percentage of these competencies did you acquire by experience?**
