# Supplementary material for: Academic pharmacist competencies in ordinary and emergency situations: content validation and pilot description in Lebanese academia
Source: BMC Med Educ. 2023 Oct 6;23:737. doi: 10.1186/s12909-023-04712-4 (PMC10559412; doi:10.1186/s12909-023-04712-4)
Supplement: Supplementary file 2 — Additional file 2. [file 12909_2023_4712_MOESM2_ESM.pdf]

Advanced Competencies for Research Pharmacists

Dear pharmacist,

You are invited to participate in a survey about advanced competencies and skills acquired upon graduation of your highest degree related to your current field of work.

This study conducted by a group of academic researchers aims to determine the domains that need strengthening for an optimal-performing public health system.

Your participation in this study is voluntary and anonymous, and the information gathered in this 20-minute questionnaire will be treated confidentially. By completing it, you are consenting to participate in this study.

We thank you in advance for your time,

The research team.

Informed consent

Please check all the boxes to proceed to the survey

☐ I have read and understood the above information

☐ I understand that my participation is voluntary

☐ I understand that my data will be kept confidential

☐ I agree to participate in this study

DEMOGRAPHICS

1. **Age:**
2. **Gender:** ☐M ☐F
3. **Level of education:**
   ☐BS Pharmacy ☐PharmD/DPharm ☐Masters ☐PhD ☐Other:
4. **Highest degree related to your main field of work:**
   ☐BS Pharmacy ☐PharmD/DPharm ☐Masters ☐PhD ☐Other:
5. **Year of graduation from school/faculty of pharmacy:**
6. **University you graduated from as a pharmacist:**
   ☐UL ☐USJ ☐BAU ☐LAU ☐LIU ☐Other, country:
7. **University you earned your highest degree from:**
   ☐UL ☐USJ ☐BAU ☐LAU ☐AUB ☐LIU ☐Other, country:
8. **What is your specialty?**
9. **Language of pharmacy education:**
   ☐French ☐English ☐Other:
10. **Work Location:**
    ☐Beirut ☐Mount Lebanon ☐North Lebanon ☐South Lebanon ☐Beqaa
    ☐Currently not working
11. **Number of working days per week:**
12. **Number of working hours per day:**
13. **How long (in years) have you been a research pharmacist?**
14. **Do you have another field of work? (Please select all that apply)**
    ☐I do not have another field of work
    ☐Academia (teaching)
    ☐Community pharmacy
    ☐Hospital pharmacy
    ☐Clinical pharmacy
    ☐Clinical preceptor
    ☐Research
    ☐Industry
    ☐Sales/Marketing
    ☐Other:

RESEARCH PHARMACIST COMPETENCIES

QUESTION: How confident are you in applying the below researcher competencies?	Very confident	Fairly confident	Neither/ I don't know	Slightly confident	Not confident at all
**0 General Research & Soft Skills**	**0.1 Critical thinking/Scientific Inquisitiveness/Ethical Thinking and Research Conduct/Strategic Thinking**				
**0.1.1** Search for, read, and criticize scientific articles.					
**0.1.2** Identify gaps in the literature.					
**0.1.3** Generate relevant biomedical, clinical, public health or translational research hypothesis.					
**0.1.4** Dedicate time to read and improve one's knowledge.					
**0.1.5** Describe the current state of knowledge about a specific topic.					
**0.1.6** Apply the ethical research principles in one's activity.					
**0.1.7** Design proper research strategies and policies.					
**0.1.8** Translate research discoveries into meaningful changes in human health.					
**0.1.9** Identify key ethical and methodological issues impacting research design and validity, including statistical techniques.					
**0.1.10** Demonstrate knowledge of the standards of professional and ethical conduct to protect human subjects enrolled in clinical research.					
**0.1.11** Identify ethical and professional issues associated to any type of research: conflict of interest, plagiarism, authorship, and intellectual property.					
**0 General Research Skills**	**0.2 Written and Oral Communication/ Grantsmanship**				
**0.2.1** Write adequate project proposals (to apply for IRB and grants).					
**0.2.2** Defend a written research proposal.					
**0.2.3** Prepare and deliver oral and written scientific information.					
**0.2.4** Communicate the results of their work adequately.					
**0 Soft Skills**	**0.3 Interprofessional Collaboration & Teamwork/Leadership**				
**0.3.1** Function in a multidisciplinary team.					
**0.3.2** Supervise others undertaking research.					
**0.3.3** Lead research teams and research units successfully.					
**0.3.4** Collaborate with others as needed to conduct and present research findings.					

**0 Soft Skills**	**0.4 Project Management, Planning, and Time Management**				
**0.4.1** Plan and manage human and financial resources of research projects.					
**0.4.2** Retrieve relevant information/evidence timely.					
**0.4.3** Evaluate, prevent, and solve possible problems in research projects.					
**0 Soft Skills**	**0.5 Information Technology**				
**0.5.1** Use information technology for research purposes.					
**1 Expertise in Fundamental Science**	**1.1 Basic Science Expertise**				
**1.1.1** Acquire expert knowledge about biological and chemical processes related to pharmaceutical sciences.					
**1.1.2** Master a scholarly field related to a fundamental research topic.					
**1 Expertise in Fundamental Science**	**1.2 Analysis Tool Expertise**				
**1.2.1** Acquire expert knowledge about analytical processes related to pharmaceutical sciences.					
**1 Expertise in Fundamental Science**	**1.3 Expertise in Conducting Fundamental Research**				
**1.3.1** Generate mechanistic hypotheses and predictions based on prior evidence.					
**1.3.2** Plan specific experimental procedures that test particular predictions.					
**1.3.3** Gather data via experimentation.					
**1.3.4** Analyze and interpret data appropriately.					
**1 Expertise in Fundamental Science**	**1.4 Drug Development Process Expertise**				
**1.4.1** Describe and contribute to drug development processes.					
**2 Expertise in Interventional Trials**	**2.1 Translational/Clinical Expertise**				
**2.1.1** Master a scholarly field related to a clinical/translational research topic.					
**2.1.2** Assess the clinical implications of scientific information.					
**2.1.3** Argue in favor of the clinical implications of a given research hypothesis.					
**2 Expertise in Interventional Trials**	**2.2 Analysis Tool Expertise**				
**2.2.1** Acquire expert knowledge on analyses related to clinical/translational research.					
**2.2.2** Analyze and interpret data appropriately.					
**2.2.3** Identify and discuss research study limitations.					
**2.2.4** Present research findings, including any limitations to their interpretation and use.					
**2 Expertise in Interventional Trials**	**2.3 Expertise in Conducting Clinical/Translational Research**				
**2.3.1** Conceive and perform appropriate clinical/translational studies related to research hypotheses.					

**2.3.2** Develop appropriate methods to recruit and retain study participants for a selected research design.					
**2.3.3** Generate hypotheses and predictions based on prior evidence.					
**2.3.4** Plan specific interventional procedures that test particular predictions.					
**2.3.5** Gather data via interventions.					
**2.3.6** Generate a plan for data security and management.					
**2.3.7** Identify essential outcomes measures to include in patient-oriented clinical trial designs.					
**2.3.8** Develop appropriate conclusions based on results from research data.					
**2.3.9** Develop an approach to overcome barriers in translating research to humans.					
**3 Expertise in Applied Pharmacy Fields**	**3.1 Generating Epidemiological and Pharmaco-Epidemiological Data**				
**3.1.1** Conduct and evaluate observational studies critically:					
**3.1.1.1** Generate hypotheses from preexisting data.					
**3.1.1.2** Conceive and realize studies in pharmaco-epidemiology and pharmacy practice.					
**3.1.1.3** Develop appropriate methods to recruit and retain study participants for a selected observational research design.					
**3.1.1.4** Identify essential outcomes measures to include in observational research.					
**3.1.1.5** Generate a plan for data security and management.					
**3.1.1.6** Define bias in research.					
**3.1.1.7** Minimize biases and maximize causality concept evidence.					
**3 Expertise in Applied Pharmacy Fields**	**3.2 Contribution to Disease Surveillance, Drug Errors, and Side Effects Surveillance**				
**3.2.1** Apply epidemiological surveillance techniques.					
**3.2.2** Apply pharmacovigilance techniques.					
**3 Expertise in Applied Pharmacy Fields**	**3.3 Validation of Measurement Tools (particularly those related to pharmacy and public health)**				
**3.3.1** Conceive and validate measurement tools on a given population.					
**3 Expertise in Applied Pharmacy Fields**	**3.4 Analysis of Associations Between Potential Risk Factors and Health Status**				
**3.4.1** Select the appropriate statistical approach for the interpretation of preclinical and clinical datasets.					
**3.4.2** Analyze data using advanced statistical methods.					

**3 Expertise in Applied Pharmacy Fields**	**3.5 Comparison of Therapeutic Methods Effectiveness**				
**3.5.1** Design and perform meta-analyses.					
**3 Expertise in Applied Pharmacy Fields**	**3.6 Answering Controversial Therapeutic Matters**				
**3.6.1** Apply principles of evidence-based medicine in judgment and research practice.					
**3 Expertise in Applied Pharmacy Fields**	**3.7 Contribution to the Elaboration of Therapeutic Recommendations**				
**3.7.1** Develop a therapeutic protocol/guideline for medications-related issues or management.					
**3.7.2** Argue in favor of the clinical/public health implications of a given research hypothesis.					
**4 Professional Development**					
**4.1** Engage in regular professional development activities					
**4.2** Engage in professional organization activities					
**5 Pharmacist Emergency Preparedness and Response (EPR)**	**5.1 Emergency Preparedness and Response**				
**5.1.1** Check for volunteering opportunities					
**5.1.2** Check for training opportunities					
**5.1.3** Follow actions and recommendations of local authorities					
**5.1.4** Include students, staff, and resources from educational institutions in research activity					
**5 Pharmacist Preparedness and Response in Emergency Situations**	**5.2 Operations Management**				
**5.2.1** Develop workplace training and safety protocols (e.g., social distancing)					
**5.2.2** Secure PPEs or other needed materials, when applicable					
**5.2.3** Monitor workers/assistants for symptoms					
**5.2.4** Adapt working hours to meet essential services during crises					
**5.2.5** Secure sanitizers when needed					
**5.2.6** Answer EPR related calls					
**5.2.7** Participate in interdisciplinary training to EPR teams					
**5 Pharmacist Preparedness and Response in Emergency Situations**	**5.3 Population Health Research**				
**5.3.1** Address medication reviews, panic buying, screening and/or testing/vaccination services safely in research activity					
**5.3.2** Focus on at-risk populations in research activity					
**5.1.3** Address medication shortage and mitigation plan in research activity					
**5.1.4** Address stockpile and availability of drugs for existing/chronic conditions in research activity					
**5.1.5** Partner with local authorities in research activity					

5 Pharmacist Preparedness and Response in Emergency Situations	5.4 Evaluation, Research, and Dissemination for Impact and Outcomes				
**5.4.1** Lead in research and studies on EPR					
**5.4.2** Publish and/or disseminate findings					
**5.4.3** Combat misinformation by disseminating evidence-based information to patients and sharing it on social media					
**5.4.4** Develop training programs to peers and other healthcare workers					

1. **What percentage of these competencies did you acquire during your undergraduate studies?**
2. **What percentage of these competencies did you acquire during your postgraduate studies?**
3. **What percentage of these competencies did you acquire from continuing education sessions?**
4. **What percentage of these competencies did you acquire by experience?**
