# Supplementary material for: Academic pharmacist competencies in ordinary and emergency situations: content validation and pilot description in Lebanese academia
Source: BMC Med Educ. 2023 Oct 6;23:737. doi: 10.1186/s12909-023-04712-4 (PMC10559412; doi:10.1186/s12909-023-04712-4)
Supplement: Supplementary file 3 — Additional file 3. [file 12909_2023_4712_MOESM3_ESM.pdf]

Advanced Competencies for Clinical Preceptor Pharmacists

Dear pharmacist,

You are invited to participate in a survey about advanced competencies and skills acquired upon graduation of your highest degree related to your current field of work.

This study conducted by a group of academic researchers aims to determine the domains that need strengthening for an optimal-performing public health system.

Your participation in this study is voluntary and anonymous, and the information gathered in this 20-minute questionnaire will be treated confidentially. By completing it, you are consenting to participate in this study.

We thank you in advance for your time,

The research team.

Informed consent

Please check all the boxes to proceed to the survey

☐ I have read and understood the above information

☐ I understand that my participation is voluntary

☐ I understand that my data will be kept confidential

☐ I agree to participate in this study

DEMOGRAPHICS

1. **Age:**
2. **Gender:** ☐M ☐F
3. **Level of education:**
   ☐BS Pharmacy ☐PharmD/DPharm ☐Masters ☐PhD ☐Other:
4. **Highest degree related to your main field of work:**
   ☐BS Pharmacy ☐PharmD/DPharm ☐Masters ☐PhD ☐Other:
5. **Year of graduation from school/faculty of pharmacy:**
6. **University you graduated from as a pharmacist:**
   ☐UL ☐USJ ☐BAU ☐LAU ☐LIU ☐Other, country:
7. **University you earned your highest degree from:**
   ☐UL ☐USJ ☐BAU ☐LAU ☐AUB ☐LIU ☐Other, country:
8. **What is your specialty?**
9. **Language of pharmacy education:**
   ☐French ☐English ☐Other:
10. **Work Location:**
    ☐Beirut ☐Mount Lebanon ☐North Lebanon ☐South Lebanon ☐Beqaa
    ☐Currently not working
11. **Number of working days per week:**
12. **Number of working hours per day:**
13. **How long (in years) have you been practicing as a clinical preceptor?**
14. **Do you have another field of work? (Please select all that apply)**
    ☐ I do not have another field of work
    ☐Academia (teaching)
    ☐Research
    ☐Other:

CLINICAL PRECEPTOR COMPETENCIES

QUESTION:<br><br>How confident are you regarding the below clinical preceptor competencies?	Very confident	Fairly confident	Neither/ I don't know	Slightly confident	Not confident at all
**0 Professional Development**	**0.1 Ethical and Professional Skills**				
**0.1.1** Combine an empathetic and supportive attitude toward students while maintaining an unwavering commitment to excellence in patient care.					
**0.1.2** Combine a commitment to continuous quality improvement with reflection on action to support meaningful learning experiences.					
**0.1.3** Ensure that the pharmacy environment embodies the same principles of practice that they hope their student pharmacists will embrace.					
**0.1.4** Identify own areas for improvement and be willing to openly discuss any issues or events that need improvement as a learning experience for themselves and their students.					
**0.1.5** Demonstrate a caring attitude toward students and patients.					
**0.1.6** Discuss the rationale used in practice with students to make sure they understand specific behaviors.					
**0.1.7** Commit to their organization, professional societies, and the community.					
**0.1.8** Engage in regular professional development activities					
**0.1.9** Engage in professional organization activities					
**1 Supervising and Teaching Skills**	**1.1 Effective Clinical Teaching Skills**				
**1.1.1** Assess learning opportunities for the students.					
**1.1.2** Give the students responsibilities for patient care while still monitoring their actions in a non-threatening way.					
**1.1.3** Demonstrate procedures.					
**1.1.4** Provide opportunities to do procedures.					
**1.1.5** Observe student performance frequently.					
**1.1.6** Correct students without belittling them.					
**1.1.7** Provide supplementary learning activities, when needed, to achieve all.					
**1 Supervising and Teaching Skills**	**1.2 Responsible Teaching**				
**1.2.1** Be responsible for the actions of their students.					
**2 Facilitating Learning**	**2.1 Coaching skills, listening skills, positive feedback**				
**2.1.1** Empower students to ask questions, search for answers, and become independent learners.					

**2.1.2** Question students thoughtfully and encourage feedback.					
**2.1.3** Identify and respond to each student specific learning needs.					
**2.1.4** Challenge the learning process in each student.					
**2.1.5** Coach student behavior through effective and timely feedback.					
**2.1.6** Prioritize student teaching as a primary focus of the practice site.					
**2.1.7** Treat students as colleagues in training.					
**2.1.8** Inspire students to develop patient care practices.					
**2 Facilitating Learning**	**2.2 Active Learning Facilitation**				
**2.2.1** Engage students in the process of active learning and good communication.					
**2.2.2** Help students to become life-long learners.					
**2.2.3** Encourage active participation in a group.					
**2.2.4** Establish a harmonious relationship with students based on understanding.					
**2.2.5** Show personal interest in students.					
**2.2.6** Facilitate the development of critical thinking, problem solving and decision-making in students.					
**3 Clarity, Organizational/Time Management Skills, and Enthusiasm**	**3.1 Organization Skills**				
**3.1.1** Explain clearly.					
**3.1.2** Present material in an organized manner.					
**3.1.3** Summarize information.					
**3.1.4** Communicate what is expected to be learned.					
**3.1.5** Teach by example.					
**3.1.6** Answer questions carefully and precisely.					
**3.1.7** Balance clinical and teaching responsibilities.					
**3.1.8** Commit to continuous quality improvement for managerial techniques, clinical skills, and practice sites to support the professionalism of pharmacy practice.					
**3 Clarity, Organizational/Time Management Skills, and Enthusiasm**	**3.2 Rules and Regulations**				
**3.2.1** Follow rules and regulations set by the program of experiential activities.					
**3.2.2** Schedule and plan activities such as case discussions, journal club, topic presentations, and other activities set by the program.					
**3.2.3** Document and assess student performance.					
**3 Clarity, Organizational/Time Management Skills, and Enthusiasm**	**3.3 Time Management**				
**3.3.1** Manage their own time well, manage their students' time, and teach students to manage it efficiently.					

**3.3.2** Use tools that can help manage time for students and preceptors.					
**3.3.3** Allocate activities through the rotation effectively.					
**3.3.4** Educate students about the role that time management plays in the financial health of any practice setting.					
**4 Interpersonal/Communication Skills**	**4.1 Communication Skills**				
**4.1.1** Interact and communicate effectively with administrators, healthcare colleagues, peers, patients, or students to reach desired outcomes.					
**4.1.2** Develop an effective student-preceptor relationship.					
**4.1.3** Be willing to listen to the students and learn something new.					
**4.1.4** Convey confidence in students' abilities to allow them to work harder to reach goals.					
**4.1.5** Provide positive reinforcement and constructive feedback so that students can understand their strengths and improve their weaknesses.					
**4.1.6** Possess conflict management skills and be prepared to teach students to use them.					
**4.1.7** Use public speaking skills to speak effectively in large and small group situations.					
**4.1.8** Provide effective medication and onsite-related education training/presentations to students, pharmacists, and other healthcare professionals.					
**5 Cultural Competency Skills**	**5.1 Cultural Competency Skills**				
**5.1.1** Provide care to patients with diverse values, beliefs, and behaviors, including tailoring delivery to meet patients' social, cultural, and linguistic needs.					
**5.1.2** Appreciate and value cultural differences to provide high-quality care to patients from diverse backgrounds.					
**5.1.3** Respect and value these differences among students and collaborate in a manner that promotes education.					
**5.1.4** Strive to foster an attitude of cultural competence in their students.					
**5.1.5** Encourage student pharmacists to accept and respect differences.					
**5.1.6** Create an open and trusting environment for communication with people from diverse cultures.					
**5.1.7** Have the will to self-assess their performance in working with patients from differing backgrounds.					
**5.1.8** Provide students with resources, such as links to websites that support cultural competency and diversity to help expand their cultural knowledge.					
**6 Leadership Skills**	**6.1 Leadership Skills**				
**6.1.1** Demonstrate effective managerial and leadership relationships with pharmacist colleagues and staff.					

**6.1.2** Demonstrate aspects of humility related to own limitations.					
**6.1.3** Monitor quality of professional practice and teaching activities.					
**6.1.4** Evaluate and refine the practice and activities on an ongoing basis to continually improve their activities in the pursuit of excellence.					
**6.1.5** Practice non-discriminatory behaviors.					
**6.1.6** Embody a personal practice philosophy.					
**6.1.7** Demonstrate personal motivation and inspiring students and pharmacists to develop patient care practices.					
**6.1.8** Have a mission or vision statement of pharmacy.					
**6.1.9** Encourage administration to support patientcare service.					
**6 Leadership Skills**	**6.2 Motivation**				
**6.2.1** Have the desire to educate others (patients, caregivers, other healthcare professionals, students, pharmacy residents).					
**6.2.2** Demonstrate enjoyment and enthusiasm for patient care and teaching.					
**6.2.3** Adapt a stimulating style of presentation to capture student's attention.					
**6.2.4** Stimulate interest in the subject and motivate students to learn.					
**7 Knowledge and Clinical Skills**	**7.1 Clinical Skills**				
**7.1.1** Possess clinical skills and use them in practice.					
**7 Knowledge and Clinical Skills**	**7.2 Other Technical Skills**				
**7.2.1** Use effective educational techniques in the design of all educational activities.					
**7.2.2** Use information technology to make decisions and reduce errors.					
**8 Research and Project Management Skills**	**8.1 Research & Project Management Skills**				
**8.1.1** Participate in a practice-related research project using effective project management skills.					
**8.1.2** Encourage students to present and publish their work on projects.					
**9 Pharmacist Emergency Preparedness and Response (EPR)**	**9.1 Emergency Preparedness and Response**				
**9.1.1** Check for volunteering opportunities					
**9.1.2** Check for training opportunities					
**9.1.3** Address medication shortage and mitigation plan with students and peers					
**9.1.4** Facilitate the educational institution partnering with local authorities					
**9.1.5** Check for FDA/EMA Emergency Use Authorizations (EUAs) and expedited review and approval of tests/drugs for treatment					
**9.1.6** Follow actions and recommendations of local authorities					
